# Supplementary material for: A highly diverse, desert-like microbial biocenosis on solar panels in a Mediterranean city
Source: Sci Rep. 2016 Jul 5;6:29235. doi: 10.1038/srep29235 (PMC4932501; doi:10.1038/srep29235)
Supplement: Supplementary Information [file srep29235-s1.pdf]

A highly diverse, desert-like microbial biocenosis on solar panels in a Mediterranean city

Pedro Dorado-Morales[1,+], Cristina Vilanova[1,2,+], Juli Peretó[1,2,3], Francisco M. Codoñer[4], Daniel Ramón[5] and Manuel Porcar[1,2,*]

[1]Institut Cavanilles de Biodiversitat i Biologia Evolutiva, Universitat de València, Paterna 46980, Spain

[2]Darwin Bioprospecting Excellence, SL., Parc Cientific Universitat de València, Paterna, Valencia, Spain

[3]Departament de Bioquímica i Biologia Molecular, Universitat de València, Burjassot 46100, Spain

[4]Lifesequencing S.L., Parc Científic Universitat de València, Paterna, Spain.

[5]Biopolis S.L., Parc Cientific Universitat de València, Paterna, Valencia, Spain

*Address correspondence to Manuel Porcar: manuel.porcar@uv.es

[+]These authors contributed equally to this work.

Supplementary information

Supplementary Tables

Table S1. Taxonomical classification of the bacterial species isolated from solar panel samples according to 16S rDNA sequence similarity.

#	Isolate ID	Best BLAST hit	#	Isolate ID	Best BLAST hit
1	S10-LB-01	*Planomicrobium glaciei*	28	S13-LB-03	*Kocuria rosea*
2	S10-LB-02	*Kocuria rosea*	29	S13-LB-04	*Planomicrobium chinense*
3	S10-LB-03	*Kocuria rosea*	30	S13-LB-05	*Planomicrobium koreense*
4	S10-LB-04	*Microbacterium paraoxydans*	31	S13-LB-06	*Pseudomonas psychrotolerans*

5	S10-LB-05	*Planomicrobium glaciei*	32	S13-MAR-01	N.A.
6	S10-LB-06	*Arthrobacter agilis*	33	S13-MAR-02	*Bacillus aquimaris*
7	S10-LB-07	*Kocuria sediminis*	34	S13-MAR-03	N.A.
8	S10-LB-09	*Kocuria rosea*	35	S13-MAR-04	*Planococcaceae bacterium*
9	S11-LB-01	*Kocuria rosea*	36	S13-MAR-05	N.A.
10	S11-LB-02	*Micrococcus xinjiangensis*	37	S13-MAR-06	*Planomicrobium okeanokoites*
11	S11-LB-03	*Planomicrobium glaciei*	38	S14-LB-01	*Planococcaceae bacterium*
12	S11-LB-04-01	*Bacillus infantis*	39	S14-LB-02	N.A.
13	S11-LB-04-02	*Bacillus infantis*	40	S14-LB-03	*Planomicrobium chinense*
14	S11-LB-05	N.A.	41	S14-LB-04	*Kocuria rosea*
15	S12-LB-01	*Kocuria rosea*	42	S14-MAR-01	*Planomicrobium okeanokoites*
16	S12-LB-02	*Cellulosimicrobium cellulans*	43	S14-MAR-02	*Planomicrobium okeanokoites*
17	S12-LB-03	*Planomicrobium glaciei*	44	S14-MAR-03	N.A.
18	S12-LB-04	*Enterobacter cloacae*	45	S15-LB-01	*Kocuria rosea*
19	S12-LB-05	*Kocuria rosea*	46	S16-LB-01	*Kocuria rosea*
20	S12-LB-06	*Bacillus flexus*	47	S16-LB-02	*Arthrobacter agilis*
21	S12-LB-07	N.A.	48	S16-LB-03	N.A.
22	S12-LB-08	*Planomicrobium glaciei*	49	S17-LB-01	*Kocuria rosea*
23	S12-LB-09	*Planomicrobium glaciei*	50	S18-MAR-01	N.A.
24	S12-LB-10	*Cellulosimicrobium cellulans*	51	S18-MAR-02	*Planomicrobium chinense*
25	S12-LB-11	*Kocuria rosea*	52	S18-MAR-03	*Domibacillus robiginosus*
26	S13-LB-01	*Planomicrobium okeanokoites*	53	S19-MAR-01	*Domibacillus robiginosus*
27	S13-LB-02	*Planomicrobium okeanokoites*			

N.A.: Non-assigned. Low quality sequences or assignations

Table S2. Summary of sequencing statistics from the 16S/18S profile analysis.

	Solar panel pool (2013)		Solar panel 1 (2014)		Solar panel 2 (2014)		Solar panel 3 (2014)	
	16S	18S	16S	18S	16S	18S	16S	18S
# Sequences obtained	485094	105781	47013	4566	48633	5446	25971	18260
Average length	582	574	495	456	498	478	505	507
Total Mb	282,32	60,71	23,28	2,08	24,26	2,61	13,13	9,27
# Sequences after trimming	136194	6463	25184	796	23628	1722	14804	6220
Averag. length after trimming	549	551	473	475	481	478	486	490
# Genera (>0.01% abundance)	160	40	119	43	168	34	138	28
# Species (>0.01% abundance)	303	49	249	78	343	63	271	61
Shannon index	4,94	4,01	5,64	3,74	6,23	4,89	5,31	4,62

Table S3. Summary of sequencing statistics from the shotgun metagenomic sequencing of solar panels 1 and 3 in 2014.

	Solar panel 1 (2014)	Solar panel 3 (2014)
Number of reads obtained	18596346	19220250
Average length	186,315	186,345
Total Mb	3464,783	3581,649
Average quality	32,855	32,995
Number of sequences after trimming	18240690	18716970
Average length after trimming	188,19	188,81
Total Mb after trimming	3432,688	3533,922
Average quality after trimming	33,075	33,275
Number of contigs after assembly	1486634	717211
N50	229	335
Maximum contig length	6408	13796
Average contig length	262	405
Number of predicted ORFs	104311	189676
Number of genera (>0.01% abundance) [1]	254	453
Number of species (>0.01% abundance) [1]	487	669
Shannon index [2]	4,39	5,57

[1] Estimated from BLASTP searches of the predicted ORFs against the NCBI nr database (e-value < 1e-5)
[2] Calculated from species-level assignations

Table S4. Differentially expressed proteins between day- and night-collected samples.

		Day/Night t-value	p-value	Fold Change Day/Night	Fold Change Night/Day
**gi|504554779**	cold-shock protein [Geodermatophilaceae]	3,122	0,035	25,986	0,038
**gi|656266558**	molecular chaperone GroEL [Arsenicicoccus bolidensis]	4,361	0,012	13,665	0,073
**gi|503987093**	50S ribosomal protein L7/L12 [Niastella koreensis]	3,041	0,038	9,944	0,101
**gi|502016435**	hypothetical protein [Deinococcus deserti]	3,625	0,022	9,373	0,107
**gi|517310793**	ATP-1 H+-transporting ATP synthase [Fusarium fujikuroi IMI 58289]	-3,263	0,031	0,473	2,114
**gi|656340823**	S-layer protein [Deinococcus sp. RL]	-5,991	0,004	0,346	2,890
**gi|636360381**	major outer membrane lipoprotein 1 [Klebsiella pneumoniae MGH 64]	-2,847	0,047	0,189	5,280
**gi|657196340**	50S ribosomal protein L7 [Acidiphilium angustum]	-3,031	0,039	0,188	5,326
**gi|499563314**	F0F1 ATP synthase subunit beta [Synechococcus elongatus]	-2,968	0,041	0,150	6,686
**gi|493585871**	diguanylate cyclase [Frankia sp. EUN1f]	-2,884	0,045	0,116	8,630
**gi|398394263**	isocitrate dehydrogenase [NAD] sub 1 [Zymoseptoria tritici IPO323]	-16,097	0,000	0,066	15,147
**gi|518290483**	molecular chaperone GroEL [Roseomonas]	-9,372	0,001	0,057	17,594
**gi|618851123**	membrane-bound proton-translocating pyrophosphatase [Clostridium tetanomorphum DSM665]	-3,028	0,039	0,024	40,965

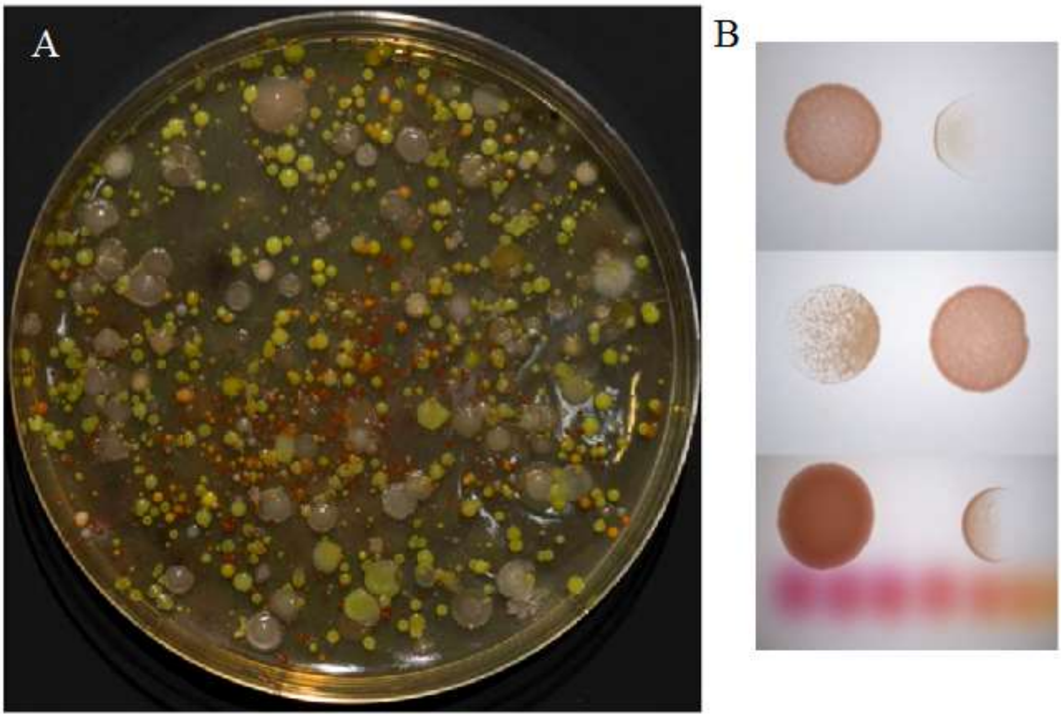

Figure S1. Microbial colonies growing on LB incubated at room temperature for two weeks (**A**). Case example of growth restoration in nearby-grown isolates under conditions of extreme pH. A local buffering of the pH of the plate is observed (**B**).

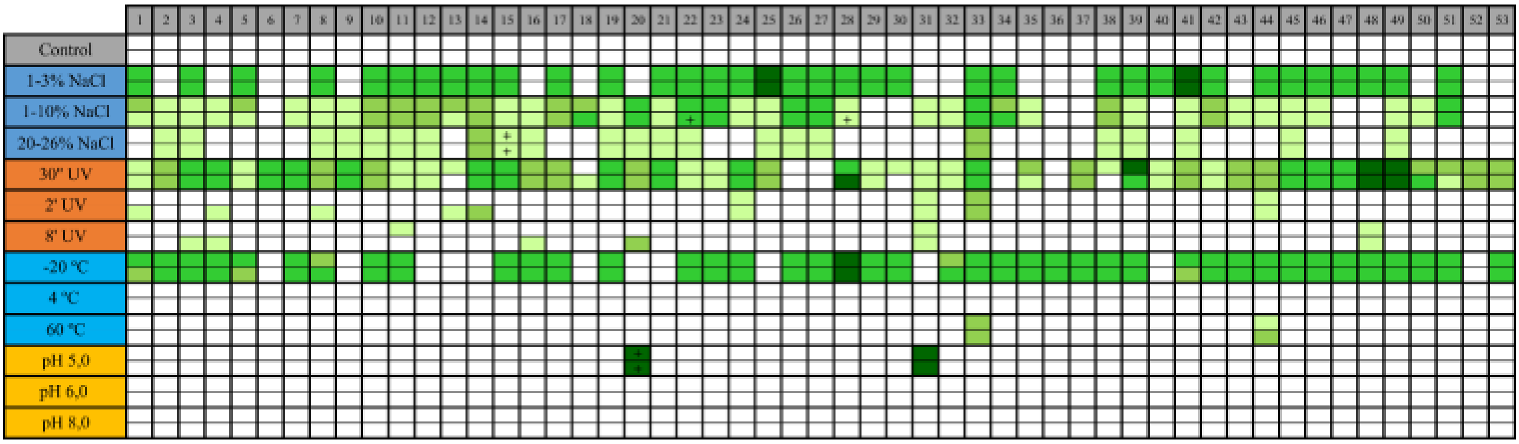

Figure S2. Stress tests results data matrix. The growth of the 53 strains isolated from the solar panels under particular stress conditions was compared to that of a control strain (XL1-Blue *E. coli* strain). Green colour (from light to dark) indicates better growth than the control (from slightly to strongly better growth). Symbols '+' indicate cases of growth restoration by another isolate. Two independent experiments were performed for each test. Strains are numbered according to Table S1.

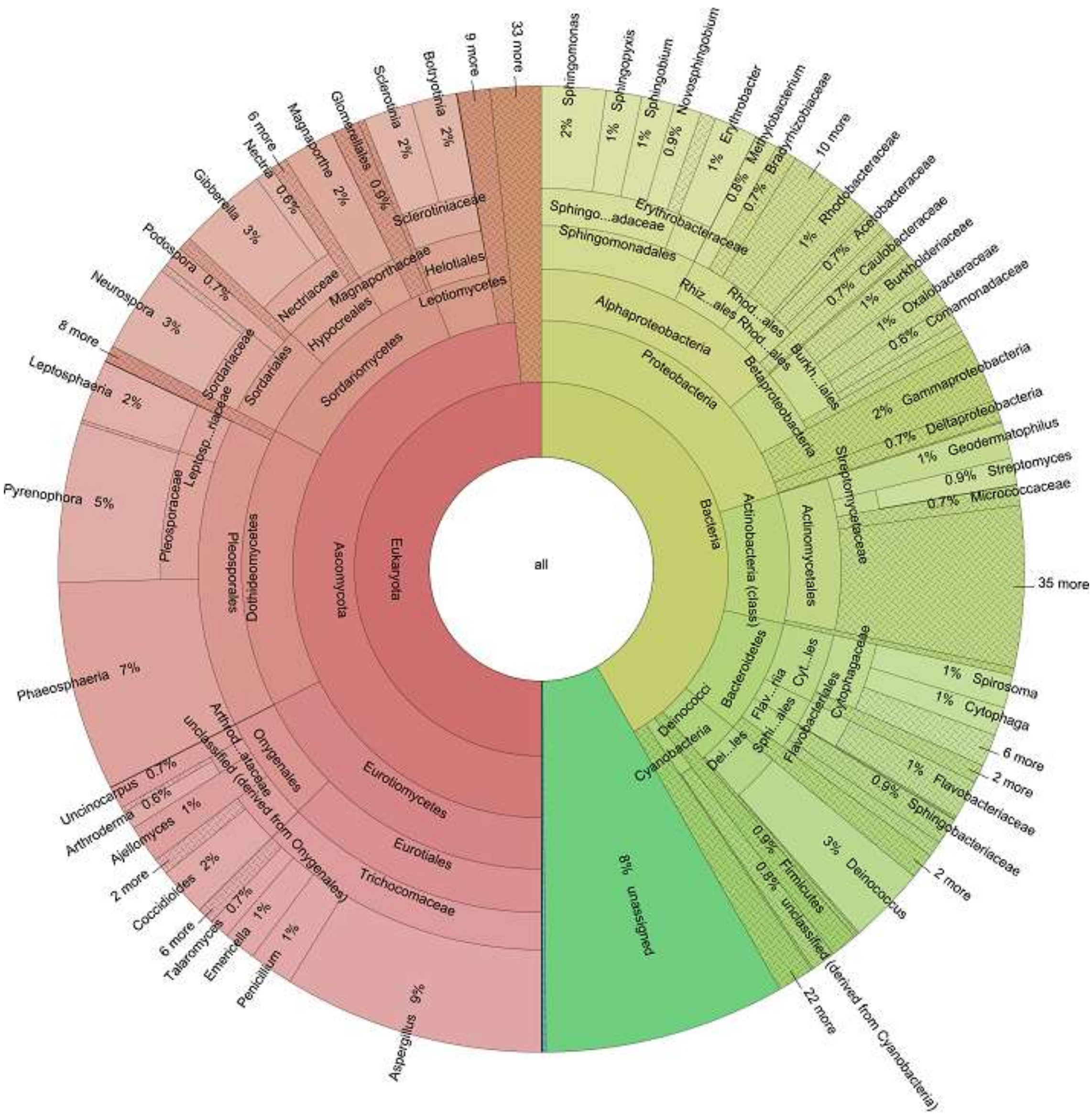

Figure S3. Taxonomic diversity of one of the panels (panel 1) sampled in the summer solstice of 2014 as deduced from shotgun metagenomic sequencing.
